# The Temporality of Situated Cognition

**DOI:** 10.3389/fpsyg.2020.546212

**Published:** 2020-09-29

**Authors:** David H. V. Vogel, Mathis Jording, Christian Kupke, Kai Vogeley

**Affiliations:** ^1^Research Center Jülich, Institute of Neuroscience and Medicine (INM3), Jülich, Germany; ^2^Department of Psychiatry, Faculty of Medicine and University Hospital Cologne, University of Cologne, Cologne, Germany; ^3^Department of Psychiatry, Society for Philosophy and Sciences of the Psyche, Charité, Humboldt-University Berlin, Berlin, Germany

**Keywords:** temporality, time experience, dynamic systems, duration (time), cognitive processing, psychopathology

## Abstract

Situated cognition embeds perceptions, thoughts, and behavior within the contextual framework of so-called “4E cognition” understanding cognition to be embodied, enactive, extended, and embedded. Whereas this definition is primarily based on the spatial properties of a situation, it neglects a fundamental constituent: the cognitive situation as *enduring*. On a subpersonal level, situated cognition requires the integration of information processing within a minimal temporal extension generating the basic building blocks of perception and action (“microlayer” of time). On a personal level, lived situations and experienced narratives leading to our biography can be defined by their broader temporal horizons (“macrolayer” of time). The macrolayer of time is based on and emerges from information processing on the microlayer of time. Whereas the constraints on the microlayer are primarily defined by the integrity of neurobiological processes within an individual cognitive system, the temporal horizons and subsequently the situational context on the macrolayer are defined by the complex affordances of a situation on a personal or interpersonal level. On both time layers, cognition can be defined as a continuous dynamic process, reflecting the transition from one situated state to another. Taken together, the events forming the delimiting horizons of these situations correspond to the temporal structure of the cognitive process along which it continuously proceeds. The dynamic driving and enabling this transition from state to state is synonymous with the inherent flow of time. Just as the layers of time, flow and structure, are inseparably connected. The integration of temporal flow and temporal structure into the continuous dynamic process constitutes the enduring situatedness of cognition. By providing everyday examples and examples from psychopathology, we highlight the benefits of understanding cognitive processes as part of enduring situations.

## Introduction

The proposal of situated cognition views cognitive processes as inherently located within a contextual framework comprising of a bodily (embodied), affording (enacted), physical (extended), and sociocultural (embedded) environment, also referred to as 4E cognition (Newen et al., 2018; Overmann and Malafouris, 2018). As the categories of 4E cognition are by definition intertwined and inseparable from the processes they constitute, situated cognition attempts at examining, interpreting, and explaining the processes underlying cognition within situations defined by these categories. As an example, consider playing a musical instrument. The music is most likely influenced by the bodily state of the player such as, e.g., emotional states. In playing, these emotions as well as the production of the tones are embodied in the musician and automatically translated into bodily functions not primarily necessary for the act of playing (e.g., rocking movements, facial expressions; embodied). Further, the musician needs to be able to hear and evaluate their own playing of the instrument adaptively (e.g., the music is too loud because the keys are being hit too forcefully), or if playing in front of an audience may want to respond to the reactions of the audience and change the corresponding manipulation of the instrument accordingly (enacted). The musician may use an instrument to play and sheet music to remember a song (extended). And lastly, the musical genre and the instrument of choice may be influenced by social–cultural determinants and biographical influences (embedded). These four antecedents to situated cognition are often conceptualized as necessary preconditions to (human) cognition (e.g., Anderson, 2003; Niedenthal et al., 2005; Smith and Gasser, 2005; Bickhard, 2008; Gallagher, 2009). Although it is theoretically possible, cognitive processes, as they appear to us, do not happen without a physical form (embodied), are not entirely undirected (enacted), and do not appear without a physical (extended) or sociocultural (embedded) context. It has, however, been argued that aspects of a given cognitive act theoretically may be reduced to a point at which it is temporally unextended (Gallagher, 2000; Zahn et al., 2008; Arzy and Schacter, 2019). These conceptions run the danger of identifying the capacities of the cognizer purely by spatial properties. Cognition is subsequently located in a purely spatial *situs cogitans*. This site of thought can then be reidentified as the spatial relationships around or within it, or as the contents of the space surrounding and included in the *situs cogitans*. This reduction theoretically constrains all four aspects of situated cognition: embodiment is reduced to the concept of a cognitive process as placed in a location, which coincidentally is a body or a brain; enaction is minimized to a simple nonrecurring computation of inputs and outputs; extendedness refers to a merely spatial distribution of cognitive subfunctions and not explicitly to temporal extension; finally embeddedness may simply correspond to geometric relationships between the cognizer and their surroundings. Against the backdrop of cognition as situated 4E cognition, such an understanding of cognitive processes as timeless is hard to uphold and threatens the integrative understanding of cognitive processes. Reconsidering our example of playing a musical instrument, it is of course obvious that no aspect of it can be solely spatial. This leads to our proposal that temporality is necessarily a part of all aspects of a situated cognitive process. It is *embodied* in our lived experiences (Menary, 2008), *embedded* into our historical past (Kupke, 2009); it is it is *extended* in space, to be stored for later use or for us to be manipulated through present, future directed *enaction* (Gallagher et al., 2017).

As an alternative example, consider solving a simple math problem (Wilson and Clark, 2009). As an equation indicates a mathematical fact such as “34 × 12 = 408,” it can by itself be conceptualized as non-temporal. It could hence be argued that cognitive representation of the equation also may be situated within a non-temporal (purely spatial) *situs cogitans*. This would effectively imply recognizing the content of the equation as an objective fact standing outside of time. However, such a conception would not fit with how we usually interact with intellectual problems. Just as performing music, the cognition leading to the mathematical equation is a lived temporal act, too. The knowledge and abilities required to solve the equation depend on an enduring cognitive procedure with 4E properties. First, we (automatically) perform motor actions corresponding to the calculation, such as counting with our fingers, or experience bodily feelings such as frustration with related motor expressions in, e.g., facial expressions (*embodied*). Second, we type in individual numbers into the calculator or write down the equation in a specific way; in other words, we identify subaspects of the math problem and use them to manipulate our surroundings in order to solve the problem (*enacted*). Third, we may use aids such as pen and paper or a calculator to solve the problem (*extended*). Fourth and finally, the math problem solver is only presented with the problem and able to understand and deal with it because cognition is *embedded* into a sociocultural context. Despite our ability to potentially recognize, e.g., mathematical facts as outside of time and space, the cognitive act of performing the mathematical operations, as well as the cognitive acts of perceiving, understanding, and solving/interacting with such facts never are. In the following, we will lay out that any (cognitive) act must by default not only be conceptualized as extended, enacted, embodied, and embedded in space but also needs to be considered as enduring over time, necessitating temporality.

Temporality as the experience of and in time has long been identified as a key component of human existence and experience (e.g., Kant, 1781, p. 28ff; Husserl, 1928, p. 384ff). However, time is often spatialized (e.g., “time windows,” “time distance,” “time lines,” etc.) and thus reduced to events and measures such as seconds or dates (Bergson, 1920, p. 82ff). Such conduct neglects the central role of temporality in our experience. As we pursue activities and carry out actions, time is ever present. While acting in the present, we enact control over our circumstances and wish to influence our (near or distant) future (Vogel et al., 2020). We do so by making use of past experiences and acquired knowledge. As such, our present can never be restricted to a “now” in the sense of an abstract point in time (Heidegger, 1953a, p. 406ff; Ricoeur, 1980). The present simultaneously includes a variety of temporal references and is necessarily a part of our ongoing narrative as an extended period of time. Time and temporality provide our actions with meaning and purpose regardless of their contents. Accordingly, any cognitive situation is not only spatially expanded but also temporally enduring. It is not merely a spatially expanded *situs cogitans*, but a temporally enduring situated cognition.

The research on social cognition has not been oblivious to temporality (e.g., Gallagher, 2009), and it is important to recognize the implications of temporality of situated cognition, which would otherwise run the danger of being overlooked. Despite cognition implicitly and necessarily involving temporality, time is not of a trivial nature. Just as cognition is implicitly and necessarily embodied, enacted, extended, and embedded, it is temporally enduring. We believe that the characterization of cognition as a “dynamic process” (Clancey, 2008) accounts best for this specific feature of the temporality of situated cognition. To avoid the reduction of the cognizer to a *situs cogitans*, we will elaborate on these temporal principles of situated cognition and will illustrate its relevance for psychopathological disturbances. In order to avoid the misconception of the *situs cogitans*, we will first elaborate on the layers of time on which and through which cognition situates itself. Second, we will demonstrate how cognition is composed of a dynamic procedure, which determines the enduring situation. Furthermore, we will argue that the experience of time and duration reflect the abilities of the cognizer to successfully reach their goal. Finally, we will illustrate the relevance of temporality for psychopathological disturbances. To this end, we will attempt to draw from different theoretical approaches. We wish to demonstrate that despite differing and in some cases opposing ideas from these approaches (see Grush, 2006; Piekarski, 2017; Zahavi, 2018; for discussion), they may converge in important parts of their content and be put into a productive dialogue pushing the boundaries of current theory.

## The Macrolayer and the Microlayer of Time

Time is of interest to us on two levels. We call the first phenomenally accessible level the macrolayer of time, or the biographic–personal time (Kupke, 2009, 2020; Vogel et al., 2020). The macrolayer describes time as it is open to conscious experience. This means both time as passing, for example, fast or slow, as well as our biographic narrative structuring past experiences and future plans. If we speak about situations on the macrolayer of time, we mean all temporal aspects consciously recognizable by a cognizer and of relevance to the currently ongoing cognitive process. This definition is not restricted to consciously, e.g., remembered or anticipated events, but refers to all potentially relevant aspects of an ongoing act, both explicitly and implicitly in the corresponding situation—both known and unknown to the cognizer independent from whether they have explicitly become aware of it or not.

Similar to the spatial aspects of a situation, the temporal features of a situation are defined by its borders, or “horizons” (Husserl, 1928, e.g., pp. 402, 411; Ricoeur, 1988; Gallagher et al., 2017). We argue that these borders define the present situation of a cognizer. For seemingly any action or activity, the horizons are foremost given by the reference points of beginning and end. Actions and steps performed in a situation are directed at the end of the situation starting from its beginning. At the horizons of a musical performance may, for example, stand the moments during which the first and the last notes were played. However, the temporal situation may extend beyond that specific period of the musical performance in the strict sense. In our example of the musician, depending on the context of the play and the context we as the observers with our own subjective point of view are interested in, the horizons of the situation may vary. For the musician’s performance, the play may have started, e.g., when coming on stage for an audience, and it ended when it was left. Further, the performance may be part of an even wider situation, such as the musician’s career opportunities and how they view themselves in relation to these opportunities.

Alternatively, we might not even be interested in the entire performance. We may want to observe the occurrence of a particular motif or theme in a piece of music. It then might be helpful to define horizons in terms of the movements of the musician to produce the motif or theme. By doing so, we identify a smaller duration within the subjective present. However, the way in which that particular motif is being played again may depend on its further temporal context, e.g., its position in the piece. Accordingly, at almost any graspable time scale, it would be possible to identify both larger and smaller time scales related to it. Although the observed individual need not be aware of this multitude of preconscious temporal horizons, they may all affect the execution of their play, as well as their observation.

This generalizes to any temporal situation. In the mathematical example, when the equation “34 × 12 = 408” is presented to us on a piece of paper, the perception of the written numbers is not a singular event. In terms of an ongoing situated cognition, it is *embodied* in our lived experiences (Menary, 2008) as we have learned how to count and calculate in school; it is *extended* in space for us to be manipulated through future directed *enaction* (Gallagher et al., 2017) as we use our capacities to understand or validate the equation, and it is *embedded* into our historical past (Kupke, 2009) with math and numbers as historical derivatives. Hence, the situation is by default *enduring*. Importantly, these aspects of the cognitive act do not need to be explicitly known by the cognizer; they are, however, necessarily implicitly present in any cognitive act.

Theoretically, we are always able to pause, identify our present situation, and reflect upon it both in smaller and larger units. Human beings seem effortlessly able to perform this reflective task and position themselves and their actions in their individual time containing both a past and a future. Simultaneously, this potential overview on their own derived narrative (Ricoeur, 1980; Stanghellini and Mancini, 2017, p. 56f; Vogel et al., 2020) remains ever present, even while not consciously being aware of it. As we will elaborate in more detail below, the cognitive act is made possible by the constitution of the cognizer as continuously enduring along the structure of time.

These considerations of the potentially experienceable phenomenon hint at a related distinction between the *implicit* experience in time and the *explicit* experience of time. Time on the macrolayer is not always directly experienced, but remains in the background while we live our lives (Fuchs, 2005, 2013; Vogel et al., 2020). This implicit experience in time is observable in a variety of temporal phenomena, such as habit (Howell, 2015; Fuchs, 2018), corporeality/embodiment (Fuchs, 2005; Wehrle, 2020), historical circumstances (Kupke, 2009), and intersubjectivity/synchronicity (Fuchs, 2005, 2013; Bloch et al., 2019) [also see von Gebsattel, 1954a, p. 137f for a similar distinction between experienced time (“erlebter Zeit”) and lived time (“gelebter Zeit”)]. However, this implicit temporality on the macrolayer of time is still potentially consciously accessible for us through either reflection or may impose on us under certain circumstances, as we will demonstrate in more detail in the section *Enduring Situatedness*.

When considering shorter temporal horizons, we notice that at some point of temporal reduction any smaller durations are no longer directly experienceable because the time interval that separates the corresponding horizons has become too short and is no longer open to conscious experience (Vogel et al., 2020). No later than then have we reached the microlayer or temporal–intentional layer of time (Vogeley and Kupke, 2007; Kupke, 2009). The microlayer of time is by definition not available to conscious experience but describes temporality as a necessary prerequisite to cognitive processing. To avoid confusion with the implicit temporality on the macrolayer of time, we will refer to the subpersonal microlayer processes as *intrinsic* temporality (Lenzo and Gallagher, 2020), although the terms sometimes have been used synonymously (Fuchs, 2013; Vogel and Vogeley, 2020).

The microlayer can be described by biological and (neuro-)physiological processes and phenomenological approaches. From a phenomenological perspective, the quintessential thoughts of Edmund Husserl are the most influential with respect to temporal consciousness (Husserl, 1928; Kupke, 2009). As an example, Varela (1999) has proposed that the diachronic unity of self-consciousness (Vogeley and Kupke, 2007) is achieved through the reciprocating oscillations of neural cell assemblies. Furthermore, Bayesian predictive processing recently has been used to account for the phenomenon of enduring consciousness (Hohwy et al., 2016; Wiese, 2017). Lastly, ongoing motor activity can be described in terms of motor cognitive models, such as, e.g., the forward model (Wolpert, 1997; Gallagher, 2000). In motor cognition, such models reflect the preconscious cognitive process of, e.g., performing a voluntary motor action.

Despite some of these theories and analyses being primarily directed at the concept of consciousness, whereas others concern cognition, they are all targeted at the same principle. More importantly, the underlying observation that temporal continuity is essential and inherent to the basic functioning of perception and experience is shared. Not only due to these similarities and convergences between the theories from different perspectives, we believe it essential to recognize that the operations on this level of time necessitate temporality. Because of its procedural nature, any such process is made up of consecutive, sometimes parallel and reemerging steps. These steps are taken by means of an inherent drive advancing and changing the content and condition of the cognitive process. As these changes occur over time, and because of their sequential and contingent nature, a dynamic process is not conceivable outside of time, but only in time.

As on the macrolayer, cognitive processes on the microlayer follow 4E properties including and in addition to their enduring quality. As taking place within a biophysical system, they are embodied; as goal-directed processes, they are enacted; as including peripheral and external information, they are extended and embedded. Finally, as continuously transitioning from one state to the next, relying on prior and expected states, the cognitive process on the microlayer is also to be characterized as enduring. In the math problem example, despite being able to appreciate the equation as a whole, we first need to appreciate every single digit, construct the numbers, understand the symbols, and derive the corresponding conclusions. Most of this process happens automatically, and only subsequently is the impression of the equation as a whole facilitated by the underlying microlayer processes. Importantly, any cognitively derived conclusion will need to undergo processing within the minimally enduring cognitive act.

Conclusively, this means that the horizon of each situation is contextually different both across acting individuals and observers. What may be defined as a present situation is highly dependent on the perspective of the cognizing and acting individual, as well as the observer’s perspective. However, the overall form of a situation as a temporally extended and enduring (inter)subjective present that in turn is both composed of smaller temporal units and embedded into a larger biographical context cannot be ignored.

## Dynamic Procedures—The Flow and Structure of Time

For any situation to be defined as temporal, it needs to be enduring and to take time. As we have argued in the previous section, temporal extendedness is a necessary condition for a cognitive process that claims to entail 4E properties. In order to integrate the multitude of perceptual information with prior knowledge and form a productive output, any cognitive mechanism needs time (Pöppel, 1997). Concerning the brain, this seeming triviality is first a biological and a physical property. In order to pass information from, e.g., the retina to the visual cortex a multitude of complex neurophysiological processes need to take place (Varela, 1999). Molecules need to change their configurations; action potentials have to be generated; transmitters are being released, traverse the synaptic cleft, and bind to receptors of the postsynaptic membrane of another neuron, from where new action potentials depart, etc. Just as the cognitive processes that these biochemical processes relate to, they are not only a localizable fact inside the neural system, but also follow temporal orders (Varela, 1999; Vogeley and Kupke, 2007). Not surprisingly, the time these processes necessarily take influences the temporal resolution of perception. Accordingly, as the neural processes take different amounts of time, the temporal resolution of different perceptual modalities differs too (Pöppel, 1997). Despite these perceptual limitations, our experiences appear to us as continuous (Husserl, 1928). Although it is open to debate whether the flow of consciousness is in fact continuous or only appears as such (e.g., Dainton, 2002; White, 2018), two observations seem obvious: (i) time moves toward the future and constitutes a passage; and (ii) time is organized along events that compose a structure (Kupke, 2009; Vogel et al., 2020).

It is important to keep in mind that despite the seemingly objective nature of these observations, with our approach we cannot make any deeper claim as to the nature of “objective time,” be it biological, physical, or ontological in nature. The temporal situatedness as described herein primarily addresses temporality as it appears to subjective experience and cognition. Although we may believe the objective and the subjective to be intricately connected (Zahavi, 2018) when we propose that time appears as in motion, we mean that experiences are *felt* as moving into and toward the future. It seems impossible to stop time in its passage and transform experiences into a truly timeless nature. We experience our stream of perceptions and thoughts from a first-person point of view as being in a constant change of “pure transition” (Kupke and Vogeley, 2009, Kupke, 2020), and even states described as timeless have at least one thing in common with any other state: they end and turn into a different state.

If we try to explain cognitive states with the term “situated cognition” and apply this principle of ongoing experience, we reach the definition of the “dynamic process.” The term “dynamic” or “transitional process” adequately describes the temporal properties of situatedness. Per definition processes are composed of stages of action, and in the case of the situated cognitive process, the cognizer cognizes through these stages. This process of cognizing necessarily implies a transition from one stage to the next to achieve an insight or a thought as the result of cognition. By virtue of the dynamic movement within the ongoing process, there is inevitable change in any such system. Additionally, it is not only the resulting cognitive state that is subject to change. Depending on the given affordances, the process itself may be altered in order to better address the context. Accordingly, we observe the dynamic in at least two forms of transition enabling continuous changes. One is the transition on the microlayer of time. Perceptions and impressions are replaced by the appearance of a following perception or impression. Integrative mechanisms within the brain receive and operate changing inputs. On a neurobiological level, action potentials and neural oscillations produce new events of the same type. The entire microlayer seems to follow the overall anisotropy of time itself (Vogel et al., 2020).

The second transition is observable on the macrolayer. When playing a piece of music, the sequential notes and the corresponding manipulations of the musical instrument need to be performed consecutively. The transition from one situation to the next is made up by a forward-directed movement that changes its content within one situational context (e.g., note after note within the piece of music). Accordingly, situations are composed of changing steps and in turn are themselves subject to change within the overarching biographical narrative.

Concerning time being structured, we notice that with the dynamic flow of time events necessarily follow one another. This pertains to the states of ongoing situations and therefore necessarily applies to their respective temporal horizons. A situation can only be properly delimited by an earlier and a later horizon if these horizons follow each other in a sensible order. We mostly structure our experiences along this temporal order of delineated meaningful events. It is further noteworthy that this structure is not restricted to past events but extends into the future by means of planning.

As with the passage of time, the structure appears both on the microlayer and the macrolayer of time (Kupke, 2009; Vogel et al., 2020). For the microlayer, Husserl’s analysis of time consciousness (Husserl, 1928) examines the way in which our consciousness is continuously constituted by a process of retention, primal impression (“Urimpression”), and protention. Impressions describe the percept as appearing to/in consciousness at the border between retention and protention: retention entails the past impressions, and protention the coming impressions. Taken together, these structural components passively synthesize (Husserl, 1928) the appearance of an enduring continuous experience. Interestingly, Husserl proposes “horizons” to retention and protention (Husserl, 1928, e.g., pp. 402, 411) behind which the too-long past retentions slip, or too-far-ahead protentions are still hidden. We understand these horizons to effectively describe the borders of the smallest possible situation: the perceptual “now.”

During any act encompassing this smallest structural entity, the microlayer of time facilitates the emergence of larger situations by executing their defining events. This macrolayer event structure visible in the multitude of larger horizontal situations has in its basics been depicted in the previous section. Effectively, the macrolayer’s structure relates to the narrative of a situation (e.g., note A was played before note B, note C is being played now, and soon note D will be played before note E). At increasing time scales, the event structure becomes experientially increasingly coarse-grained, e.g., as a musical motif or theme, and turns into a person’s account of a situation as a life phase (e.g., I was a student, became a teacher, and retired). This underlines the introductory assumption of the equation of the personal individual situation with the individual present. Unlike the moment of the “now,” the “present” is identified as a consciously experienceable duration. It has repeatedly been argued that this “present” fluctuates along a duration of several seconds potentially corresponding to the time allocated to the integration of complex multimodal environments (for a recent discussion, see White, 2017). In other words, the minimal “present” appears to last several seconds.

As implied in the previous sections, the present situation is of a potentially variable extension interindividually, making the clear distinction difficult. This difficulty of identifying the present situation naturally stems from the relationship between structure and flow during the dynamic process. The continuous transition from “now” to “now” makes it implausible to identify the impeccable horizons of any situation. Pure transition causes the continuous emergence of a new situation with a new future horizon. Despite the obvious ability to prospectively and retrospectively identify singular events and their succession, the implicit flow of time smoothens over situations constituting a contiguous and meaningful whole.

As such, the procedural structure necessarily depends on the dynamic flow. The temporal flow first causes and enables our directedness toward the future horizon of situations. Hence, the concept of flow—at least on the macrolayer of time—may be understood as analogous to concepts such as “becoming” (“Werden”) (Straus, 1928; von Gebsattel, 1954a,b; Fuchs, 2013) and “striving” (“Streben”) (Minkowski, 1923, p. 220). Both these terms appositely recognize temporal flow as directed at something. In any situation, we are necessarily directed at something. The directedness of temporal flow at the occurrence of specific desired, planned, or anticipated events emerges as a horizon of the situation. These horizons are then identifiable as the structure of time. Accordingly, this temporal structure is a structure the cognizer (en)actively defines only by means of their own temporal flow.

In the last section, we will explain how this intricate interrelation of flow and structure is met in the enduring situation. We will further provide everyday examples and examples from psychopathology to highlight the advantages of understanding cognition as enduring.

## Enduring Situatedness

What effectively remains missing in our observations is how the continuation of experience along events may account for the experience of duration in and of a given situation. In the following, we will demonstrate how the ever-present fusion of flow and structure into one dynamic process constitutes duration. It will become clear that the emergence and experienced variance of duration within a given situation are substantially influenced by and reflects a person’s condition within his/her enduring situation.

In the last section, we saw that the term *dynamic process* appositely determines cognition as temporal flow and temporal structure. Flow and structure are not separable entities but determine each other (Vogel et al., 2020). The dynamic character engenders, coherently integrates, and joins the steps of the procedure. Simultaneously, the prospective and anticipated events of the procedure draw in the flow and give it direction. Although we may conceive a flow without specific direction, we do appreciate our own actions as directed toward something (Gallagher et al., 2017). This something consists of our environmental affordances (Gallagher, 2009; Gastelum, 2018). Again, these affordances are not thinkable without a dynamic and teleological directedness of the cognitive process toward the afforded events. Accordingly, what situations afford is already provided within their future horizons. We as individuals construct situations along these inherent potentialities. As the cognizer is a necessary part of the situation, the future horizon is not determined solely by objective conditions, but necessarily by the meaning the cognizer reaffords to the situation. Out from among all the potential prospects a situation may afford, the definite meaning is determined by the overarching temporal context as provided by the situated individual.

According to the work of Bergson (1920), this overarching temporal context is equitable with the subject as enduring. Our misconception of time as spatialized intervals and events, such as seconds, or days, hides our condition of being extended along an accumulation of these inseparable events, which in turn is the true *duration* of the individual. In our own words, the interconnected dependencies of flow and structure constitute the endurance of cognitive processes of an individual; the enduring situatedness of the cognizer is comprehensible as the maximal extension of the life situation: If we embed each situation into any hypothetical, larger situation, we eventually arrive at the “pure duration” (Bergson, 1920, p. 76f) as an overarching enduring of the cognitive process and the cognizer. This enduring process is defined and facilitated by the integration of the flow and the structure of time; in other words, the enduring process continuously flows within the structure it determines.

This integration is visible in the experience of the enduring situation itself. Importantly, though, while completely immersed in a given situation, we do not pay full attention to its enduring character; in other words, we do not notice time under usual conditions, and it remains implicit. This state has been referred to as “flow state” (Fuchs, 2005; Csikszentmihalyi et al., 2014). During such a state, the situated agent is undisturbed in their active becoming from the past toward the future, and the situation’s future horizon is freely available. In other words, the situational affordance and the cognizer’s reaffordance can be brought into agreement. It is the enduring situatedness as constituted by the interdependencies of structure and flow, which determines this agreement and allows time to remain implicitly in the background of experience. As stated above, this implicit experience in time is observable in a variety of different phenomena. However, time is consciously and explicitly experienced during a variety of different situations. These experiences and the underlying and relating cognitive processes can potentially be better understood by highlighting the temporality of situated cognition. While acting toward the future horizon in such situations, we become aware of our drive toward it. The most relatable experience may be the everyday phenomenon of boredom. A situation is boring, if the present cannot be brought into agreement with the horizon of the situated cognizer sufficiently. The person is unable to direct his/her action capacity at the desired goal. If, e.g., I wish to listen to an interesting musical performance, but the performance is not exciting enough, my overarching narrative horizons of listening to an interesting performance do not apply. Unfortunately, after I have already sat down in the audience, I cannot change the situation. If I am unable to leave the situation, I will inevitably feel bored, wishing to dedicate my time to something else (e.g., Fuchs, 2005; Elpidorou, 2018).

A second common experience is that of time pressure. The available time is known to be insuf?cient to perform an action directed at reaching a particular horizon and time appears as too little. Obviously, these two portrayals are only exemplary, and both cases do not describe all possible versions of time pressure and boredom [for a thorough analysis of boredom (“Langeweile”), see, e.g., Heidegger, 1953b]. Nevertheless, both the case of boredom and that of time pressure—and we argue any situation during which time is explicitly experienced—have one commonality in terms of the enduring situation: We recognize the limits of the situation and consciously experience its duration. The future horizon is either too close (time pressure) or too far away (boredom). In any case, the inner capacity to act toward a desired goal is impaired by the situation and consequently become aware of the duration of the situation. In other words, when the experience of duration imposes on us (Heidegger, 1953b), it corresponds to our inability to adequately reach a desired future horizon. Instead, we arrive too late or too early. The resulting question, as to the difference between the conceivable modes of time experience caused by these two conflicts between desired and imposed temporal horizons, demonstrates that the broadened definition of the situated cognition as being *enduring* has significant implications for the cognizer and cannot be dismissed as trivial. As fully answering this question is well beyond the scope of this contribution, and it is not directly related to the general question of temporality in cognition as addressed herein, we wish to limit ourselves to the observation that the intricate interdependencies of flow and structure give rise to both the capacity of being active satisfactorily and its restrictions.

In this context of situatedness, satisfying activity relates to the opportunities available to an individual within time. Time is, as noted earlier, not a space where actions are counted as, e.g., seconds, but time is when we live and enact our enduring situation within its horizons. Our experience of being a situated agent acting in a dynamic reciprocity with our environment in space and during time accordingly describes the situatedness of the cognitive process.

## Enduring Situated Cognition and Psychopathology

An important implication of the temporality of cognitive processes lies in the study and understanding of psychopathological phenomena and mental disorders. As two examples, consider a psychotic episode in schizophrenia and a depressive episode in major depressive disorder (World Health Organization, 1993; American Psychiatric Association, 2013).

For patients with schizophrenia, disturbances in temporal processing have repeatedly been implicated (Fuchs, 2007; Vogeley and Kupke, 2007; Fletcher and Frith, 2009; Vogel D. et al., 2019). It has been proposed that a disruption or fragmentation in temporal continuity may lie at the heart of explaining psychotic symptoms. From a phenomenological point of view, it has primarily been argued that this fragmentation is due to an alteration of the future directed protention on the microlayer of time (Kupke, 2009, pp. 53–62; Fuchs, 2013; Stanghellini et al., 2016; Vogel D. H. V. et al., 2019; Vogel D. et al., 2019). In this context, protention is understood as analogous to an anticipatory process (Fuchs, 2013), which graduates future perceptions along a spectrum of probability. As protention fails in schizophrenia, new events cannot be sufficiently anticipated, leading to gaps in experience. These gaps in turn give rise to a variety of psychotic symptoms, including self-disorders, by means of an intrusion of thought or experience (Kupke, 2009, p. 55f; Fuchs, 2013; Stanghellini et al., 2016).

Relating this phenomenological view to current hypotheses from neuroscience, the cause for temporal fragmentation is hypothesized to lie in a faulty evaluation of predictions by the brain, caused by dysregulated dopamine release (Fletcher and Frith, 2009; Vogel D. H. V. et al., 2019). These inadequate predictions need to be explained by a dominant involvement of top-down processes that correlate to the development of delusional believes. The dysregulated dopaminergic activity causes the sudden emergence of aberrant affordances. In other words, otherwise meaningless objects and contexts suddenly appear as meaningful and important. This newly attributed relevance causes a directedness of the patient toward the aberrantly salient percept, which then has to be explained by reaffording them a personal meaning (i.e., delusion).

In terms of an enduring situated cognition, the situation of the cognizer during a psychotic episode is constantly disrupted by events unforeseeable and inexplicable to the cognizer. As we have seen, this idea of unpredictability is interestingly brought forth by both phenomenological approaches, as well as predictive processing hypotheses. In both cases, the structure of the cognitive procedure is fragmented primarily on the consciously inaccessible microlayer of time. However, the underlying dynamic driving the cognitive process remains intact. With still ongoing and enduring cognition the dynamic now connects the faulty procedural steps, forcing the cognizer to form beliefs and behaviors in accordance with an environment that is in constant and for the psychotic patient unpredictable change. This unpredictability hypothetically translates to any observation of the cognizer during psychosis, which effectively limits the transferability of individual behaviors to other persons with psychotic syndromes. The difference between the two approaches primarily lies in the formation of the symptom in question. Where, e.g., Fuchs (2013) suggests an intrusion of experiences into a “gap,” which had already been caused by faulty protention, for predictive processing approaches the intrusion itself—caused by aberrant salience—is the disrupting/fragmenting factor.

As our second example, depressive episodes have been described by psychopathologists investigating the experience of time as being characterized by a “disturbance of becoming” or a “blocked future” (Straus, 1928; von Gebsattel, 1954a,b; Fuchs, 2013; Stanghellini et al., 2017; Vogel et al., 2018). Symptoms such as loss of interest, decreased affective reactivity, depressed mood, and emotionlessness, as well as psychomotor retardation, have been linked to this conceptual phenomenon (Stanghellini et al., 2017; Vogel et al., 2018). Patients during major depressive episodes are thought to have lost the biologically anchored strive toward the future, rendering them unable to form emotional connections and to pursue or even form goals (Straus, 1928). Patients lose the ability to act within the present, in order to change their environment and hence their future.

Putting these observations in terms of an enduring situated cognition, the depressive state can be understood as an unending situation. The dynamic of the cognitive process has changed to a point where the transition to a new situation no longer actualizes itself. The cognitive process is still generating an ongoing situation; however, this situation has lost key features of its temporal dynamic. The interaction between this change in the temporality of the situation in connection to the social and interpersonal embeddedness has been speculated to be a considerable part of patients’ suffering by causing experiences of falling behind (Fuchs, 2013).

In both exemplary cases, these alterations in temporal experience have been described as hypothetically linked to changes in predictive processing (e.g., Kent et al., 2019; Vogel D. et al., 2019; Vogel D. H. V. et al., 2019; Vogel et al., 2020) just as temporality and predictive processing more generally have been (Grush, 2006; Hohwy et al., 2016; Wiese, 2017). Although these two approaches may not coincide in all respects [e.g., the debate between (neo-)neo-Kantian representationalism and Husserlian transcendental idealism (Zahavi, 2018), or see Grush, 2006 for an overview of difficulties and weaknesses of some integrative approaches mentioned or referred to herein], they converge on the intrinsic unfolding of temporality on what we have now called the microlayer of time. Although these two examples from psychopathology require more elaboration, they demonstrate that information processing and its disturbances can be fruitfully reanalyzed and potentially be better understood by appreciating the enduring quality of cognition. Together with our examples concerning satisfying activity, they highlight the benefits of making explicit the temporality of cognitive acts. For future consideration, we propose that these changes of the temporal experience in altered mental states can be much better understood in terms of an altered enduring situatedness. Similar approaches may exist for other disorders and mental states for which temporality has been proposed as a defining constituent (e.g., Zukauskas et al., 2009; Hohwy et al., 2016; Vogel D. et al., 2019; Vogel D. H. V. et al., 2019; Vogel et al., 2020).

## Conclusion

The enduring temporal context is constitutive of the situated character of cognition. With respect to the embodied nature, it contains the individual past in form of memories and schemata (Menary, 2008); as enacted and extended, it contains the individual future (Gallagher et al., 2017), as socioculturally embedded cognition contains the historical past (Kupke, 2009). We have argued that the time of the situated cognizer is lived both on a temporal–intentional microlayer reflecting a minimal duration of the cognitive process, and on a biographic–personal macrolayer reflecting an emerging narrative duration (Kupke, 2009; Vogel et al., 2020; also see Menary, 2008; Newen, 2018). We have further argued that situated cognition described as a dynamic process relates to the temporal properties of extended situations as being in flow, but at the same time following a structure. Lastly, we have demonstrated that the fusion of flow and structure engenders the capable and active agent and relates the experience of time to that of successful activity. It should now appear obvious that the premises of situated cognition overall describe cognition not in terms of a *situs cogitans* or site of thought, but as an enduring situation. Emphasizing the enduring quality of cognition is necessary to adequately describe the prerequisites to situated cognition, as well as the cognitive situation itself in order to foster a better understanding of cognitive acts in general, as well as during altered mental states.

## Data Availability Statement

The original contributions presented in the study are included in the article/supplementary material, further inquiries can be directed to the corresponding author/s.

## Author Contributions

DV, CK, and KV provided the concept for the manuscript. DV wrote the first draft of the manuscript. All authors participated in the discussion, development, and proofreading of the manuscript. All authors approved the final version of the manuscript.

## Conflict of Interest

The authors declare that the research was conducted in the absence of any commercial or financial relationships that could be construed as a potential conflict of interest.
